# Effect of therapeutic plasma exchange on amoxicillin, clindamycin, midazolam, and morphine pharmacokinetics in a critically ill child

**DOI:** 10.1007/s00467-025-06873-4

**Published:** 2025-07-03

**Authors:** André Yaghyazaryan, Valentina Gracchi, Sybrand W. J. Zielhuis, Elisabeth H. Schölvinck, Daan J. Touw, Martin C. J. Kneyber, Paola Mian

**Affiliations:** 1 Department of Clinical Pharmacy and Pharmacology, University Medical Center Groningen and University of Groningen, Hanzeplein 1, Groningen, GZ 9713 the Netherlands; 2 Division of Pediatric Nephrology, Department of Pediatrics, Beatrix Children’s Hospital Groningen, University Medical Center Groningen, University of Groningen, Hanzeplein 1, Groningen, GZ 9713 The Netherlands; 3 Department of Pediatric Infectious Diseases, Beatrix Children’s Hospital, University Medical Center Groningen, University of Groningen, Hanzeplein 1, Groningen, GZ 9713 The Netherlands; 4 Department of Pharmaceutical Analysis, Groningen Research Institute for Pharmacy, University of Groningen, Hanzeplein 1, Groningen, GZ 9713 The Netherlands; 5 Division of Pediatric Critical Care Medicine, Department of Pediatrics, Beatrix Children’s Hospital Groningen, University Medical Center Groningen, University of Groningen, Hanzeplein 1, Groningen, GZ 9713 The Netherlands

**Keywords:** Antibiotics, Pharmacokinetics, Sedatives, Therapeutic drug monitoring, Therapeutic plasma exchange

## Abstract

We report a 15-year-old boy with severe septic shock and multiple organ failure, requiring intensive care and extracorporeal therapies, including veno-arterial extracorporeal membrane oxygenation, continuous veno-venous hemodiafiltration, and therapeutic plasma exchange. Given their critical role in the clinical management, pharmacokinetics of amoxicillin, clindamycin, midazolam, and morphine were closely monitored. Drug concentrations were measured before, during, and after therapeutic plasma exchange sessions. Despite theoretical predictions, clinically significant reductions in plasma drug concentrations of amoxicillin, clindamycin, and midazolam were observed following therapeutic plasma exchange. This case report emphasizes the necessity of therapeutic drug monitoring to adjust dosing appropriately and optimize patient outcomes.

## Case presentation

A previously healthy 15-year-old boy (body weight 60 kg, height 180 cm, body surface area 1.75 m^2^) was admitted to the pediatric intensive care unit (PICU) of the University Medical Center Groningen because of a severe septic shock due to invasive Group A Streptococcus and influenza B infection. The boy had a thrombocytopenia-associated multiple organ failure, with circulatory and respiratory insufficiency, as well as an oliguric acute kidney injury (serum creatinine 196 µmol/l, estimated glomerular filtration rate according to chronic kidney disease in children (CKiD) U25 formula: 36 ml/min/1.73 m^2^). Despite intensive clinical support, including mechanical ventilation, aggressive fluid resuscitation, vasoactive medications (epinephrine, norepinephrine, and vasopressin), hydrocortisone, antibiotics (amoxicillin and clindamycin), and antiviral therapy (oseltamivir), circulatory instability persisted. Therefore, the boy was connected to a veno-arterial extracorporeal membrane oxygenation (VA-ECMO) circuit 6 h after PICU admission. Twelve hours after admission, continuous veno-venous hemodiafiltration (CVVHDF) was also started as supportive therapy for the fluid overload secondary to aggressive fluid resuscitation in combination with acute kidney injury, and for cytokine removal. CVVHDF therapy was performed through a Prismaflex^®^ System, with an ST-150 filter (Baxter, Deerfield, IL, USA). Blood flow rate was 170 ml/min. Replacement fluid was administered post-filter at a flow rate of 1000 ml/h, dialysate flow at a rate of 1000 ml/h (both as Phoxilium^®^, Baxter). Targeted ultrafiltration was 1200 ml/day. On day 2 after admission to the PICU, the child’s clinical condition was still critical, and therapeutic plasma exchange (TPE) was initiated as additional therapy [1]. TPE was performed using the Spectra Optia^®^ apheresis system with a centrifugation technique for plasma separation. The procedure was performed over 3 consecutive days, with each session exchanging 2.2 l of plasma using fresh frozen plasma. The procedure remained consistent during the three sessions. TPE was performed in tandem with ongoing ECMO and CVVHDF treatments, both of which remained unchanged during this period. The first TPE session started after the initiation of amoxicillin, clindamycin, and midazolam therapy. The second and third sessions followed on days 3 and 4 of PICU admission, with morphine added to the therapeutic regimen prior to the third session.

**Fig. 1:**
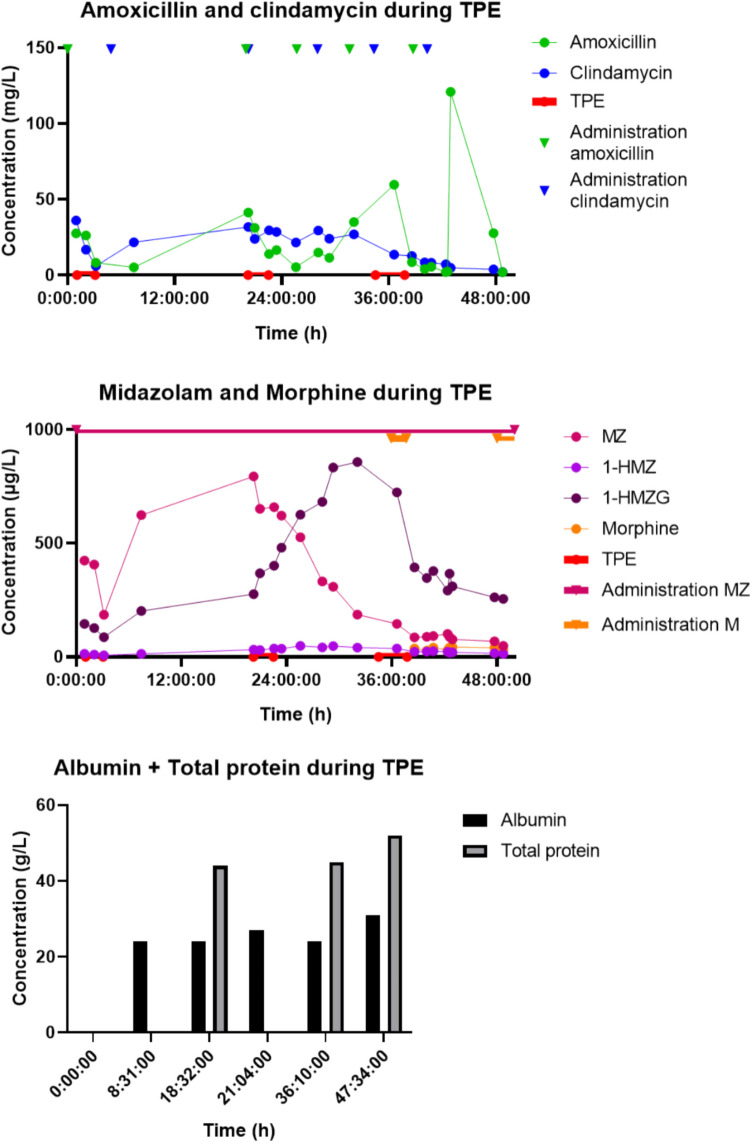
Drug concentrations of amoxicillin, clindamycin, midazolam, and its metabolites and morphine during TPE process

Given the potential impact of TPE on drug pharmacokinetics (PK) [2], concerns arose regarding the therapeutic concentrations of essential medications, including amoxicillin, clindamycin, midazolam, and morphine. Adequate drug levels were deemed critical to the ongoing treatment of this critically ill patient. Therapeutic drug monitoring (TDM) was performed to assess and optimize drug dosing in the context of TPE, the patient’s altered PK due to severe illness, and extracorporeal therapies. Blood samples for drug concentration analysis were taken before, during, and after TPE sessions to assess its impact on the PK of the administered medications. Notably, the plasma concentrations of amoxicillin, clindamycin, and midazolam decreased to a greater extent than anticipated after the TPE sessions (Fig. 1).

After three TPE sessions, the patient’s condition improved dramatically, and he could be disconnected from VA-ECMO and CVVHDF. Seven days after admission, he was discharged to the general pediatric ward, and 2 years later he is in good clinical condition.

## Discussion

We describe a critically ill adolescent with septic shock and multiorgan failure, treated with ECMO, CVVHDF, and TPE. The PK of amoxicillin, clindamycin, midazolam, and morphine were assessed given the critical role of these drugs. Literature suggests that drugs with low volume of distribution (*V*_*D*_) (< 0.2 l/kg), high plasma protein binding (> 80%), and long half-life (*t*_1/2_ > 2 h) are likely to be removed by TPE [2]. Amoxicillin has a *V*_*D*_ of 0.36 l/kg, low protein binding (17%), and a short half-life (~ 1 h). Clindamycin has a *V*_*D*_ of 0.84 l/kg, protein binding of 77%, and a half-life of 2.5 h. Midazolam has a relatively large *V*_*D*_ of 1.63 l/kg, high protein binding (97%), and a half-life of ~ 3 h. Morphine exhibits a high *V*_*D*_ of 5.31 l/kg, protein binding of 35%, and a half-life of 2.5 h [3]. Based on these properties, none of these drugs would be expected to undergo significant removal by TPE under normal circumstances.

In this context, it is important to consider that sepsis itself can significantly increase the *V*_*D*_ of hydrophilic drugs due to capillary leak and aggressive fluid resuscitation. ECMO and CVVHDF may further increase *V*_*D*_ because of drug sequestration within the circuit, hemodilution from the priming solution, and ongoing extracorporeal circulation [4, 5]. In contrast, the extracorporeal volume introduced by TPE is transient and only present during the procedure itself but may still contribute to a temporary shift in drug distribution [2].

Figure 1 illustrates the drug concentration profiles of amoxicillin, clindamycin, midazolam + its metabolites, and morphine during TPE sessions. Despite the theoretical predictions, significant reductions in the plasma concentrations of amoxicillin, clindamycin, and midazolam were observed. Amoxicillin, clindamycin, midazolam concentrations decreased by an average of 69.52% (SD = 17.98%), 42.25% (SD = 35.87%), and 31.28% (SD = 38.86%), respectively. Concentrations of midazolam metabolites: 1-hydroxymidazolam and 1-hydroxymidazolamglucoronide decreased by 7.91% (SD = 40.34%) and remained unchanged (SD = 49.85%) respectively. Morphine concentrations increased by 7.1% (Fig. 1). The variability observed over the 3 days may be attributed to fluctuations in the patient’s physiological state, variations in drug distribution due to ongoing organ dysfunction, and potential differences in TPE efficiency between sessions. Additionally, factors such as changes in plasma protein levels and hemodynamic instability could have influenced drug clearance.

Because of the concurrent ECMO and CVVHDF, the decrease in drug concentrations could theoretically be caused by any of the three performed therapies. Nevertheless, considering the clear difference in drug concentrations before and after TPE, and the fact that ECMO and CVVHDF were continuous therapies that had already been initiated and remained unchanged during the plasma exchanges, it is most likely that TPE is the contributor to observed reduced drug concentrations. Also, since TPE was performed via venous access, plasma was withdrawn after occurrence of systemic distribution. In addition, as the drugs were administered well before the start of TPE sessions, systemic distribution had already occurred. Additionally, TPE removes both the free and protein-bound fractions of drugs, whereas ECMO and CVVHDF primarily eliminate only the free fraction. Lastly, adsorption to ECMO or CVVHDF membranes is likely to have stabilized, as these systems were already in use for over 24 h before TPE was initiated. While these factors support TPE as the most probable explanation for the observed reductions, we cannot fully exclude simultaneous minor contributions from ECMO and CVVHDF. This remains a limitation and emphasizes the need for further controlled studies.

Interestingly, morphine levels were not significantly affected, possibly due to its higher *V*_*D*_, which promotes extensive tissue distribution and reduces its susceptibility to TPE removal. The increase in midazolam metabolites, observed during session two, but not during session one and three across sessions, likely reflects altered redistribution kinetics [2]. However, variability in metabolite behavior within sessions underscores the importance of cautious interpretation, particularly in small sample sizes (*n* = 3).

This case highlights the limitations of relying solely on theoretical PK predictions for extracorporeal therapies. Sepsis, critical illness, multiorgan dysfunction, and blood purifying techniques can significantly alter drug PK [3], complicating the prediction of real-world outcomes. It also emphasizes the importance of carefully planning the timing of drug administration and ongoing monitoring when using extracorporeal therapies. Clinicians must be aware of the potential for unexpected drug removal and adjust dosing regimens as needed. Furthermore, there is a critical need for well-designed studies and in vitro experiments to validate theoretical concepts, ultimately improving clinical decision-making in this area. We recommend TDM of amoxicillin, clindamycin, and midazolam concentrations in patients undergoing extracorporeal techniques including TPE to keep the drug concentrations within the therapeutic range.

## Summary

### What is new?

- Therapeutic plasma exchange can significantly impact drug pharmacokinetics, even for medications not predicted to be removed based on theoretical criteria.
- Concurrent use of multiple extracorporeal therapies in critically ill pediatric patients may require therapeutic drug monitoring to ensure optimal drug concentrations.

## Data Availability

Data is available upon reasonable request via de corresponding author.
